# Recent Developments in Materials for Physical Hydrogen Storage: A Review

**DOI:** 10.3390/ma17030666

**Published:** 2024-01-29

**Authors:** Thi Hoa Le, Minsoo P. Kim, Chan Ho Park, Quang Nhat Tran

**Affiliations:** 1 Department of Chemical and Biological Engineering, Gachon University, 1342 Seongnam-daero, Sujeong-gu, Seongnam-si 13120, Republic of Korea; lehoa290792@gachon.ac.kr; 2 Department of Chemical Engineering, Sunchon National University, Suncheon 57922, Republic of Korea; mspkim@scnu.ac.kr

**Keywords:** hydrogen, physical hydrogen storage, hollow spheres, carbon-based materials, zeolites, metal–organic frameworks (MOFs)

## Abstract

The depletion of reliable energy sources and the environmental and climatic repercussions of polluting energy sources have become global challenges. Hence, many countries have adopted various renewable energy sources including hydrogen. Hydrogen is a future energy carrier in the global energy system and has the potential to produce zero carbon emissions. For the non-fossil energy sources, hydrogen and electricity are considered the dominant energy carriers for providing end-user services, because they can satisfy most of the consumer requirements. Hence, the development of both hydrogen production and storage is necessary to meet the standards of a “hydrogen economy”. The physical and chemical absorption of hydrogen in solid storage materials is a promising hydrogen storage method because of the high storage and transportation performance. In this paper, physical hydrogen storage materials such as hollow spheres, carbon-based materials, zeolites, and metal–organic frameworks are reviewed. We summarize and discuss the properties, hydrogen storage densities at different temperatures and pressures, and the fabrication and modification methods of these materials. The challenges associated with these physical hydrogen storage materials are also discussed.

## 1. Introduction

Nonrenewable energy sources (fossil fuels) such as coal, oil, and natural gas have been playing an essential role in the global economy for over 150 years. Fossil fuels account for approximately 80% of the global energy demand [1]. However, fossil-fuel sources are expected to be exhausted and will have to be replenished. In addition, the main component of fossil fuels is carbon; therefore, when fossil fuels are burned to generate energy, carbon dioxide is released into the atmosphere, which causes various environmental and climate problems. The rapid growth of the population and economy has resulted in the fast exhaustion of fossil-fuel sources and serious environmental and climate impacts [2,3]. Therefore, governments of many countries are in a race to transform to alternative and renewable energy to secure their own energy sources and contribute to the “net-zero emission” commitment (nZEC). By 2020, more than 110 countries had signed up to achieve the nZEC target by 2050 [4]; China, one of the countries emitting large amounts of carbon dioxides, had committed to achieve this by 2060 [5]. These countries must have a “net zero-energy building” (nZEB) by 2050, which implies that the amount of energy used by a building every year must be equal to the amount of renewable energy generated onsite. “Zero emission” or “carbon neutrality” imply that even though carbon is emitted, the emitted carbon gas is captured and stored elsewhere or utilized for some purpose [6,7]. The use of renewable energy combined with carbon capture–utilization systems can help address the issues of energy deficiency and global warming.

Hydrogen is light, energy-dense, and storable, and it can be produced from fossil fuels, biomass, water, or their combinations [8]. The use of hydrogen energy does not directly produce pollutants or emit greenhouse gases; therefore, hydrogen is considered one of the cleanest energy sources. Hydrogen contributes significantly to achieving zero carbon emissions and can be considered a clean, secure, and affordable energy source of the future [9].

In addition, hydrogen can promote the development and expansion of renewable energy sources such as solar and wind energy, which are not always available to match the demand. Hydrogen is one of the best options for renewable energy transfer and storage as well as the lowest-cost option for long-term electricity storage. Hydrogen and hydrogen-based fuels can transport energy from regions with solar and wind resource abundance to energy-poor regions that are thousands of kilometers away [10,11]. Therefore, many countries are implementing policies and projects that support investments in the hydrogen industry. In addition, hydrogen production and storage technologies can be improved, developed, and scaled up to allow hydrogen to be widely used in the future. 

Currently, hydrogen storage remains challenging in hydrogen energy applications. In addition to compressed and liquefied hydrogen, hydrogen storage materials play an important role in promoting widespread applications in the hydrogen industry. Compared with the compression and liquefaction approaches, storage materials can store higher-density hydrogen safely; therefore, systems using these materials can be flexibly operated. Moreover, storage materials are the most suitable for both onboard and stationary applications [12].

**Figure 1:**
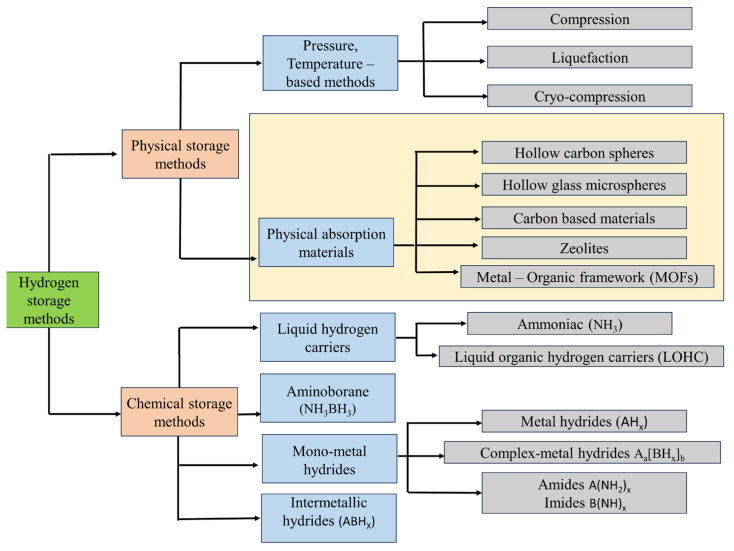
Classification of hydrogen storage materials.

Hydrogen storage materials are classified into chemisorption and physisorption types, which have pros and cons. Rationally, hydrogen storage materials need to meet the requirements of abundance, safety, low cost, rapid kinetics, easy handling, suitable thermodynamic properties (reversible hydrogen absorption and desorption), and high gravimetric and volumetric densities of hydrogen [13]. Figure 1 shows the classification of hydrogen storage materials. Chemical storage materials have potential of high energy densities and ease of use, especially liquid-involved systems using similar infrastructure to that of today’s gas-line refueling stations. However, the dehydrogenation process is relatively challenging due to several irreversible storage approaches of chemical materials [14]. It is unsuitable with ammonia and other storage compounds due to the production of gaseous pollutants such as nitrogen oxides and carbon oxides. Also, metal hydride storage materials have poor kinetics and insufficient thermodynamic stability, although they can be operated at low pressure and ambient temperature, with a higher volumetric energy density [15]. On the other hand, physical storage system can be categorized into control of conditions (pressure/temperature) and the selection of absorbents. Pressure or temperature-controlled storage systems for hydrogen gas such as cryogenic liquefaction and high-pressure compression technologies have been matured. Nevertheless, they have limitations of safety concerns, high costs, and low storage capacities [16]. The utilization of novel porous materials as a high-throughput hydrogen absorbent in physical hydrogen storage methods point to the future overcoming the critical limitation of today’s physical storage system by showing strengths of high storage densities, fast charging–discharging kinetics, and low costs [17]. In this review, we first focus on physical storage absorbents that are used to store compressed hydrogen in a hollow structure or absorb hydrogen in nano- or mesoporous structures such as metal–organic frameworks (MOFs), carbon-based materials, or zeolites.

## 2. Physical Hydrogen Storage Materials

### 2.1. Compressed Hydrogen Storage Materials

Hydrogen compression is the most widely used technology to storage and utilize hydrogen gas as an energy source with several outstanding advantages [18,19]. First, the compression technique is well developed with hydrogen filling and release occurring at high rates. Moreover, hydrogen release does not require energy [19,20]. Among the various storage materials, hollow spheres are most widely used for compressed hydrogen storage. Hollow spheres not only provide high surface area but also encapsulate hydrogen within pores. Moreover, the high surface area and porosity allow hybridization with other materials via binding sites on the surface or in the pores [21]. Hence, the absorption and catalytic activities of the hollow spheres can be increased significantly. In addition, the good mechanical strength and low specific weight of hollow spheres make them good hydrogen containers [22]. Controlling the different conditions such as reaction time, pH, temperature, and ratio of reactants can simplify the fabrication of hollow spheres of uniform sizes. In hollow nanospheres, hydrogen can be absorbed in both atomic and molecular forms [23]. 

Hollow spheres with average sizes ranging from micrometers to nanometers have attracted considerable research attention. Various types of hollow spheres with different sizes and from different materials including carbon, glass, metal, and nonmetal, have been developed. In this review, we mainly discuss hollow carbon spheres and hollow glass microspheres, which have been attracting considerable attention.

#### 2.1.1. Hollow Carbon Spheres (HCSs)

HCSs are mainly fabricated by two methods: hard templating and soft templating, which use different template types to create a hollow sphere (HS). The hard-templating approach employs special hard particles as a “core template” to build a hollow structure. The core is removed after a carbon shell is formed on the surface of the core. Silica, polymers, and hard metal particles are often used as core templates in hard-templating procedures. In soft templating, a hollow structure is directly generated by the self-assembly of carbon and other organic compounds. Hence, the core templates are “soft” precursor molecules, which easily decompose during the final pyrolysis. Table 1 illustrates the formation of HCS via hard (silica, polymer, and metal templating with the example Schemes 1–3, respectively) and soft (surfactants and organic additives templating with the example Scheme 4 and Scheme 5, respectively). Both approaches have their advantages and disadvantages. Hard templating allows better control of the HCS properties, but it requires considerable time and nonenvironmentally friendly processes to remove the core templates after the shell formation. The template is decomposed easily during soft templating [24]; however, it is more difficult to control the size and morphology of the HCS via soft templating.

HCSs are excellent materials for hydrogen storage. Their mesoporous structure provides a high Brunauer–Emmett–Teller (BET) value, voids, and hollow spaces, which can be occupied by hydrogen; thus, a high hydrogen capacity can be achieved. However, the HCS structure has a significant influence on the hydrogen storage capacity. The shape and size of the HCS play important roles in hydrogen diffusion in the substrate. When the adsorption energy is higher than the release energy, hydrogen can easily occupy the active sites that strongly depend on the morphologies of the material. In addition, the deposition of hydrogen into the pores of the HCS can be facilitated by the defects and thin walls of the HCS; hence, the spaces between the layers can be freed, increasing the amount of hydrogen stored in the HCS [30,31,32]. Hydrogen storage based on HCS has been reported in several studies, as partly presented in Table 2.

Wu et al. successfully synthesized necklace-like hollow carbon nanospheres (CNSs) with narrow pore distributions using pentagon-containing reactants. The results of their electrochemical hydrogen storage experiments showed that CNSs afforded a capacity of 242 mAh/g at a current density of 200 mA/g, corresponding to a hydrogen storage of 0.89 wt%, which is considerably greater than the electrochemical capacities of solid chain carbon spheres and multiwalled carbon nanotubes [30]. This confirms that the structure and morphology of carbon materials strongly affect the electrochemical hydrogen storage.

Doping hollow carbon materials with metals for hydrogen storage is known as hydrogen spillover [39]. Metals doped onto carbon materials can increase the binding energy between hydrogen and the pore walls. In addition, binding can occur between the hydrogen molecules and doped metallic atoms [40]. Thus, metal doping can enhance the hydrogen storage of materials. Among the various metal-doped hollow carbons used for enhancing hydrogen storage, palladium (Pd)-doped carbon materials have been reported to improve the hydrogen storage performance [41,42,43]. Hydrogen molecules easily dissociate on the Pd surface, which limits the storage efficiency. Hence, to decrease the active surface area of Pd to achieve good catalytic performance and improve the hydrogen storage capacity, small particles of Pd are doped onto the porous surface of the material. 

Michalkiewicz et al. synthesized and evaluated the hydrogen storage performance of Pd nanoparticles doped onto HCSs [41]. Their results indicated that the size and amount of Pd nanoparticles significantly affected the hydrogen storage capacity and that size played a more important role than quantity. The small size of the Pd nanoparticles could enhance their cumulative surface area, thereby increasing the spillover effect. With a diameter of 11 nm, the capacity obtained at a temperature of 40 °C and pressure of 24 bar was 0.36 wt%, which was two times higher than the storage capacity of pristine HCSs. 

Kim et al. and Jiang et al. reported Ni-loaded HCSs and N-containing HCSs, respectively. Because of their physical sorption characteristics, doping with Ni or N does not affect the hydrogen absorption at ambient temperature [44]. However, the results show that the hydrogen uptake capacities of Ni-loaded HCSs and N-containing HCSs are 1.23 wt% and 2.21 wt%, respectively, at room temperature [34,35]. This is because the dissociation of hydrogen molecules into atomic hydrogen can easily occur in the presence of Ni nanoparticles, following which hydrogen atoms are chemically adsorbed by the sorbents, resulting in an enhanced capacity for storing hydrogen. Moreover, the spillover reaction provided by Ni decoration also enhances the hydrogen storage capacity. For N-doped HCSs, N atoms can activate carriers, create more defect sites, and enhance the hydrogen–carbon binding strength on the carbon surface. Hence, the hydrogen absorption mechanism changes from physisorption to physicochemical adsorption [35,45]. 

Khaled et al. fabricated Mg ions diffused in hollow carbon nanospheres embedded with platinum nanoparticles (Mg/HCNS/Pt). This material was expected to act as an efficient hydrogen carrier for energy-release applications. At temperatures of 270, 300, 330, and 370 °C, the obtained hydrogen desorption capacities were 6.78, 6.92, 7.05, and 7.85 wt% respectively. The kinetics experiments showed that Mg/HCNS/Pt only took 85.2 and 168 s to release a maximum of 7.85 wt% at 370 °C and minimum of 6.78 wt% desorption capacity at 270 °C, respectively [37]. This study indicated that although doping with metal elements could enhance the hydrogen storage capacity of HCSs, the hydrogen adsorption capacity of this composite material was not suitable for application at ambient temperatures. Improvements in terms of new materials or fabrication techniques are necessary to overcome this limitation.

In addition to metal and nonmetal doping, the hydrogen storage capacity can be enhanced by improving the surface area of the HCS. Yang et al. used chemical vapor deposition to successfully synthesize zeolite-like HC materials with large surface areas. This material demonstrated the effect of surface area on hydrogen storage capacity, which was 2.6 wt% at a pressure of 1 bar and reached 8.33 wt% at a pressure of 20 bar. This capacity of the zeolite HC material was recorded as the highest value ever reported among HC materials.

#### 2.1.2. Hollow Glass Microspheres (HGMs)

HGMs, also known as microballoons or microbubbles, are mainly composed of borosilicate and soda lime silica. The sizes of HGMs range from 100 nm to 5 µm with a wall thickness ranging from 1.5 to 3 µm and pore size from 100 to 500 nm [46,47]. The wall thickness of HGMs strongly determines their crush strength; the higher the sphere density, the higher the crush strength. Owing to their excellent chemical stability, chemical resistance, strong mechanics, low density, high-temperature operation, high water resistance, noncombustibility, nonexplosibility, nontoxicity, and particularly, low-cost production, HGMs are considered as high-potential hydrogen transporting and storing materials [32,33,48]. 

HGMs must be operated under high pressure and high temperature to enhance the hydrogen diffusion into the HGMs. This is because not only the sizes of the gas molecules but also the temperature can change the gas diffusivity. After hydrogen molecules are absorbed in the HGMs, the temperature must be decreased to room temperature to reduce the diffusivity rate and retain the loaded hydrogen molecules in the pore cavities. For the hydrogen-release process, it is necessary to heat the HGMs to high temperatures. The optimal temperature for both hydrogen absorption and desorption is over 300 °C [49,50]. 

HGMs can be fabricated using dry-gel or liquid-droplet approaches. The high-temperature furnace in which the initial particles are formed plays an important role in both methods. First, the blowing agent is broken down to release the gas within the dried gel or liquid. The fast growth of gaseous products leads to the appearance of bubbles and the formation of hollow droplets. Thereafter, the hollow droplets rapidly cool from the liquid state to form HGMs [51]. Regarding the synthesis of HGMs, Wang et al. presented a basic formula for a new type of HGM, based on the traditional fabrication approaches, experiences in this material industry, and interrelated businesses. According to their proposal, HGM comprised quartz sand, K_2_CO_3_, Na_2_B_8_O_13_.4H_2_O, Na_2_CO_3_, Ca(OH)_2_, NaAlO_2_, Li_2_CO_3_, and H_2_O in weight percentages of 27, 14.5, 15, 10, 3.5, 3, 0.5, and 26.5%, respectively. Hence, the main chemical components of HGM were SiO_2_ (68–75%), Na_2_O (5–15%), CaO (8–15%), B_2_O_3_ (15–20%)_,_ and Al_2_O_3_ (2–3%) [52]. 

Dalai et al. presented a type of HGM for hydrogen storage, which was synthesized using an air–acetylene flame spheroidization method using urea as the blowing agent. With a microsphere diameter of 10–200 μm and wall thickness of 0.5–2 μm, they showed hydrogen storage capacity at ambient temperature and at 200 °C under a pressure of 10 bar. The results indicated that the adsorption capacity at ambient temperature was lower than that at 200 °C [53]. This confirmed that the higher the temperature, the better the gas diffusivity. At high temperatures, hydrogen molecules could pass through the HGM walls toward the hollow pores and were retained in them. 

**Figure 2:**
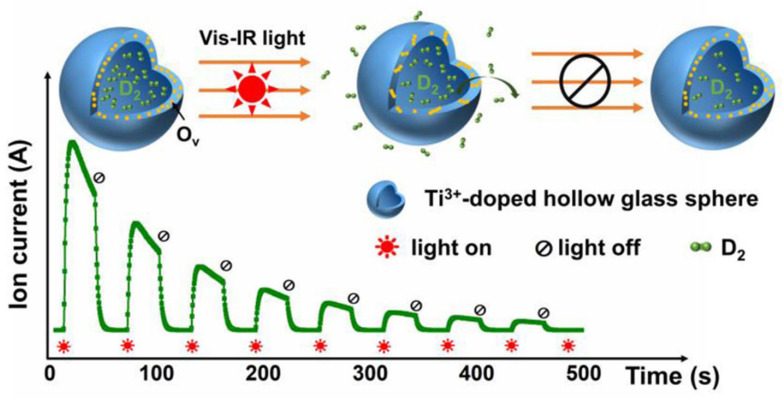
Photo-induced gas release in Ti^3+^-doped hollow glass microsphere (HGM). Reprinted with permission from [54]. Copyright 2023, Elsevier.

The poor thermal conductivity of HGMs restricts hydrogen molecule loading during the absorption and desorption processes, resulting in a poor hydrogen storage capacity [50]. It has been discovered that when HGMs are doped with photoactive agents such as Ti, Cr, V, Fe, Zn, Mg, and Co, the gas diffusion rate is strongly enhanced by light illumination compared with that of traditional heating furnace approaches [50,54,55,56]. Figure 2 illustrates photo-induced outgassing in Titanium (Ti) ion-doped HGMs due to light absorption [54]. Particularly, this research investigates the influence of Ti doping on the gas retention capacity and photo-induced outgassing behavior of HGMs. The gas retention capacity of HGMs is of great importance for hydrogen storage. In this study, the gas retention properties of HGMs with different Ti concentrations were investigated through evaluation of their pressure half-life (t1/2) and deuterium (D2) permeability. The discovery of photo-induced hydrogen diffusion through glass–shell HGMs loaded with photoactive metals paves the way for new revolutionary methods for filling, storing, transporting, and releasing hydrogen.

Dalai et al. synthesized HGM samples loaded with Fe and Mg to improve their heat transfer properties, thereby enhancing their hydrogen storage capacity. For Fe doping, the feed glass powder was mixed with certain amounts of ferrous chloride tetrahydrate solution and magnesium nitrate hexahydrate salt solution to obtain 0.2–2 wt% Fe loading and 0.2–3.0 wt% Mg loading, respectively, in the HGMs. Hydrogen adsorption experiments on all HGMs doped with Fe and Mg were performed at 10 bar and 200 °C for 5 h. The results indicated that the hydrogen uptake capacity of Fe-doped HGMs was approximately 0.56 wt% at 0.5 wt% iron loading. Increasing the iron content did not enhance the hydrogen absorption because of spheroidization and the FeO/Fe block, which prevented the accessibility of some pores. This was confirmed by the results of 0.21 wt% uptake capacity with 2 wt% Fe doping in HGMs. Meanwhile, the Mg-doped sample exhibited a hydrogen adsorption increase from 1.23 to 2.0 wt% as the Mg loading percentage increased from 0 to 2.0 wt%. This is similar to the phenomenon of Fe doping; if the amount of Mg in the HGMs was over 2 wt%, nanocrystals of MgO/Mg that could close the pores appeared, thereby decreasing hydrogen storage capacity [57]. 

**Figure 3:**
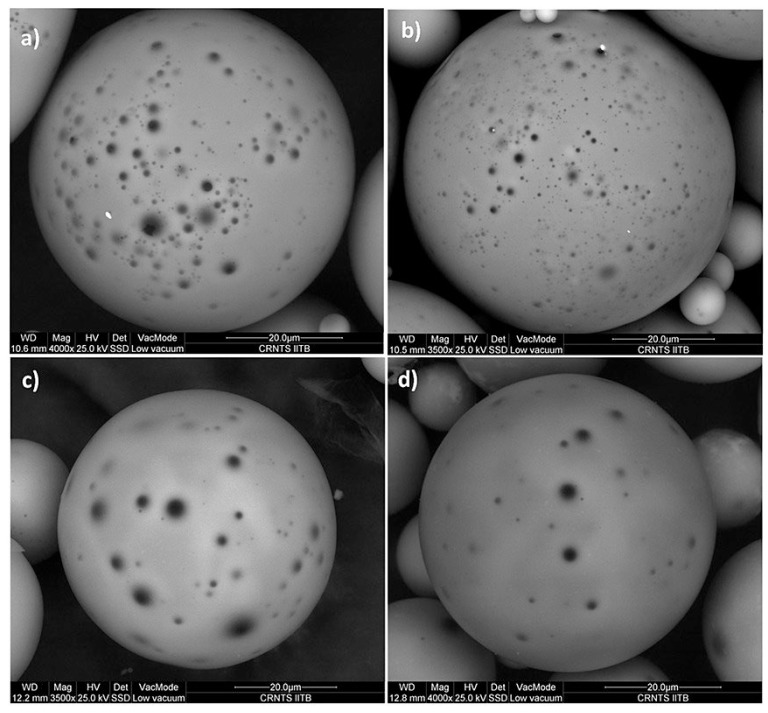
Environmental scanning electron microscopy images of HGMs doped with different amounts of zinc: (**a**) 0.5, (**b**) 2, (**c**) 5, and (**d**) 10%. Reprinted with permission from [58]. Copyright 2015, Hindawi.

Dalai et al. also fabricated HGMs loaded with Zn to improve their thermal conductivities. Figure 3 shows the environmental scanning electron microscopy images of the HGMs doped with various amounts of Zn. The results showed that Zn doping increased the thermal conductivity and pore density of HGMs. The number of pores increased when the amount of zinc rose from 0.5 wt% to 2 wt%, but it was observed that the pore width decreased. When the zinc concentration was increased to 5 wt% and 10 wt%, most of the small pores were closed, and the pore number was reduced too because the deposition of the ZnO-rich layer blocked the pores in the HGMs [58]. 

To sum up, the metal doping can increase the porosity of HGMs and then lead to an enhancement in hydrogen storage capacity. However, the too high concentrations of metal can result in the formations of metal oxide layers or the agglomeration metal particles, which can close the pores of HGMs and then decrease the hydrogen loading volume. Hence, doped-metal concentration optimization is extremely crucial to obtain the highest hydrogen storage capacity for HGMs. Overall, we summarize the general characterization of the glass microspheres in Table 3.

Co-loaded HGMs have also been fabricated [59,60]. The results revealed that the number of pores in the HGMs increased significantly and that the hydrogen storage capacity reached a maximum of approximately 2 wt% at Co concentration ≤2 wt%. When the amount of Co exceeded 2 wt%, energy-dispersive spectroscopy (EDS) analysis confirmed the formation of cobalt oxides on the walls of HGMs, which occupied the pores and prevented hydrogen diffusion. 

### 2.2. Physical Absorption Materials

The physical hydrogen absorption process involves loading hydrogen onto the material surface. The origin of this process is the resonant fluctuations in the charge distribution, which are known as dispersive forces or van der Waals interactions. The interaction forces between hydrogen molecules and other materials are quite weak; therefore, physisorption only occurs at temperatures lower than 0 °C [61]. However, because of its advantages of high energy efficiency [46], high rates of loading and unloading [46,62], and good refueling time [63], physisorption has been widely used in the recent years. Hence, absorbent materials have been developed and improved to meet the physisorption requirements. A good hydrogen absorbent depends on two factors: (1) the binding energy between hydrogen molecules and materials, which directly affects the operating temperature of the hydrogen storage system, and (2) the availability of a high average surface area per unit volume [64]. Recently, materials considered excellent candidates for hydrogen physisorption include carbon-based materials such as activated carbon (AC), carbon nanotubes (CNT), graphite nanofibers (GNF), graphene, zeolites, and MOFs, which are further discussed in the following sections.

#### 2.2.1. Carbon-Based Materials

Carbon is one of the most abundant elements in both living and nonliving organisms. Carbon materials are easily prepared as powders with high porosity and have a propensity to interact with gas molecules. Therefore, carbon is a well-known medium capable of absorbing gases and has been used as a detoxifier and purifier.

Carbon-based materials have attracted much attention as promising materials for hydrogen storage because of their several advantages such as high surface area, high porosity with diverse pore structures, good chemical stability, low weight, and low cost [65,66]. Various carbon-based materials are used for hydrogen storage. In this review, we focus on the use of materials such as AC, CNT, GNF, and graphene for hydrogen storage, as summarized in Table 4. Examples of hydrogen storage mechanisms using AC, CNT, GNF, and graphene nanocomposites are shown in Schemes 6–9, respectively (Table 5).

#### 2.2.2. Zeolites

Zeolites are three-dimensional, microporous, and highly crystalline aluminosilicate materials. Zeolites have a tetrahedral framework structure in which Si and Al atoms are tetrahedrally coordinated through shared oxygen atoms. Zeolites are well-known materials with channels and cages in which cations, water, and small molecules can reside. In addition, their high thermal stabilities and good ion-exchange capacities render them a huge potential promising media for hydrogen storage [113,114].

Zeolites can be classified as natural or synthetic. Natural zeolites exhibit improved resistivity and thermal stability in different environments [115]. However, they contain impurities that are not uniform in crystal size; therefore, they cannot be utilized in industrial applications [116]. In this review, we focus on the synthetic zeolite materials used in the hydrogen storage industry.

Zeolites can be fabricated using various raw materials. To meet the requirements of economic efficiency, the raw materials should be readily available, relatively pure, selective, and inexpensive. Raw materials such as kaolin, rice husk ash, paper sludge, fly ash, blast furnace slag, lithium slag, and municipal solid waste are mostly used for zeolite fabrication. 

Zeolites can be synthesized though different approaches including solvothermal, hydrothermal, ionothermal, alkali fusion and leaching, microwave, and ultrasound energy methods, which have been presented in previous studies [116]. Among these approaches, the hydrothermal process is the most widely used, particularly for the synthesis of zeolite membranes [117].

Owing to their excellent properties and various synthetic processes, zeolites are considered as good hydrogen storage candidates. Dong et al. investigated the hydrogen storage capacities of different zeolites including Na-LEV, H-OFF, Na-MAZ, and Li-ABW. The results showed that at a pressure of 1.6 MPa and temperature of 77 K, the capacities of Na-LEV, H-OFF, Na-MAZ, and Li-ABW were 2.07, 1.75, 1.64, and 1.02 wt%, respectively [114]. This result confirms that micropores with a higher volume and diameter, approximating the kinetic diameters of the hydrogen molecules, play a vital role in enhancing the zeolite capacity.

**Figure 4:**
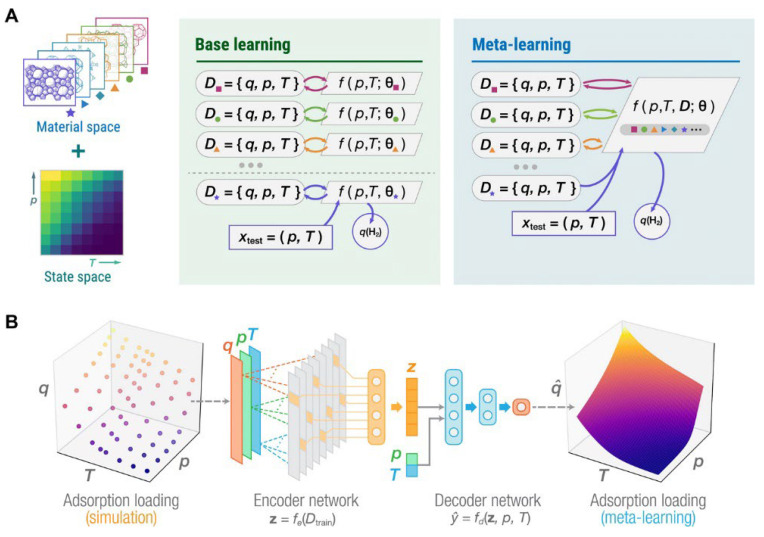
Meta-learning for predicting gas adsorption loading, *q*, in nanoporous materials. (**A**) Setup of meta-learning problem. (**B**) Meta-learning architecture for gas adsorption prediction. Reprinted with permission from [118]. Copyright 2021, American Association for the Advancement of Science.

Recently, Sun et al. developed Monte Carlo simulations to predict hydrogen loading under different pressure and temperature conditions for MOFs, hypercrosslinked polymers, and zeolites. Regarding the pressure value, meta-learning allowed the identification of the optimal temperature with the highest working capacity for hydrogen storage. Figure 4 describes the meta-learning system used for the gas uptake prediction. According to this report, the RWY-type and AWO-type zeolites exhibit a hydrogen loading capacity of approximately 7 wt% under a pressure of 100 bar and temperature of 77 K [118]. 

#### 2.2.3. MOFs

**Figure 5:**
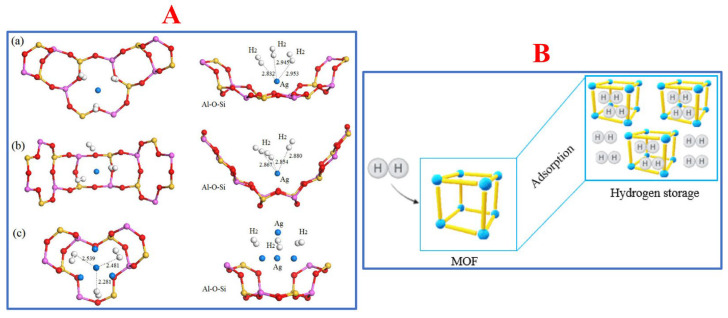
(**A**) Hydrogen adsorption of zeolites materials named (a) aAg0.29Li0.71-LSX, (b) bAg0.43Li0.57-LSX, and (c) cAg0.29Li0.71-LSX. Reprinted with permission from [122]. Copyright 2019, MDPI; (**B**) Scheme of MOFs capturing hydrogen via their adsorbent properties. Reprinted with permission from [123]. Copyright 2022, MDPI.

MOFs are an extensive class of crystalline materials that contain metal ions, metal clusters, and organic linkers. Organic linkers such as azoles (1,2,3-triazole, pyrrodiazole, etc.), dicarboxylates (glutaric acid, oxalic acid, succinic acid, malonic acid, etc.), and tricarboxylates (citric acid, trimesic acid, etc.) are commonly used in MOF fabrication [119,120]. In addition to controlling the metal/ligand ratio, changing the reaction temperature and modifying the organic linker are strategies to afford MOFs with good pore structures and high-dimensional frameworks [121], which significantly affect the hydrogen storage capacity. The main hydrogen storage mechanism of MOFs is similar to that of other porous materials such as hollow spheres, carbon-based materials, or zeolites: hydrogen molecules occupy and are kept in their vacants (the pores). The schematic mechanism examples of zeolites and MOFs are described in Figure 5.

MOFs materials are excellent candidates for potential applications in clean energy such as media for storing gases such as hydrogen and methane because of their high crystallinity, large internal surface area that can reach up to 6000 m^2^/g, and ultrahigh porosity with up to 90% free volume [124]. In addition, the variability in the metal and organic components in their structures endows MOFs with good designability, tunable structures, and properties. However, the production of MOFs is quite complex, and the hydrogen uptake efficiency of MOFs at ambient temperature is low. Therefore, improvements in the MOF fabrication technology are necessary [125].

A wide range of MOFs synthesis processes have been developed, which are summarized in Table 6. After synthesis, the MOF material obtained is typically available in a powder form. The incorporation of powders into relevant devices is quite difficult; this can prevent the utilization of MOFs in energy or gas-storage applications [126]. For example, if the MOFs are introduced as a loose powder into a tank with pipe fittings, they may be easily blown off into the surroundings, causing difficulties in handling and contamination in pipe fittings. In addition, their low packing density can compromise the volumetric capacities of the pipe fittings. Scientists have made great efforts to shape MOFs using the techniques presented in Table 6. The choice of the shaping technique depends on the fabrication approach and expected textural properties of the MOF materials [127,128]. Shaped MOF materials with appropriate mechanical strength, low flow resistance, and intact or high secondary surface areas and pore volumes are desired for better hydrogen storage performance [126].

Hydrogen molecules have low polarizability. In addition, interactions between hydrogen and most MOFs are relatively weak. Previous research showed that the isosteric heat of hydrogen absorbed in the highly porous MOFs was approximately −5 kJ/mol because of the weak interactions of hydrogen molecules [125]. Hence, cryogenic temperatures are essential for obtaining reasonable hydrogen capacities in MOFs. We conducted research on some hydrogen storage MOF materials and made a small statistic, as shown in Table 6. The results indicate that the MOF materials exhibit good hydrogen storage capability at low temperatures. Many research groups synthesized and investigated their MOF materials for hydrogen storage, such as MIL-53 (Cr) modified with Pd-loaded activated carbon (MIL-53 Cr) [129], MIL-101 (Cr) with zeolite-templated carbon ZTC (MIL-101 (Cr)/ZTC) [130], iron-based MOF (Fe-BTT) [131], and Co-based MOF (Co-MOF) [132]. They obtained hydrogen storage capacities of less than 5 wt%. With the improvements in the components and structures of materials, as well as using suitable pressures, the recent MOF materials including Zr-based MOFs (NU-1101, NU-1102, NU-1103), Al-based MOFs (NU-1501-Al) [133], and robust azolate-based MOFs (MFU-4/Li) [134] achieved remarkable increases of 9.1, 9.6, 12.6, 14, and 9.4 wt%, respectively, in hydrogen storage capacities.

However, storage at ambient temperatures remains a challenge. To overcome the limitations of the driving ranges in fuel-cell vehicles when using adsorption-based hydrogen storage technology, researchers have been developing different methods to increase the performance of MOF materials at higher temperatures. For example, open metal sites in the framework can act as strong adsorption sites for hydrogen loading at ambient temperatures. Lim et al. synthesized Be-based MOF (Be-BTB) with open metal sites. The Be-BTB material with a high BET surface area of 4400 m^2^/g presented a hydrogen adsorption capacity of 2.3 wt% at 298 K and 100 bar, which is much higher than that of the previous studies [135]. In addition, doping with metal ions or incorporating carbon-based materials may introduce a hydrogen spillover effect (HSPE) that can improve the hydrogen storage capacity of materials at room temperature. The HSPE is a surface phenomenon in which active hydrogen atoms generated by the dissociation of hydrogen molecules on metal surface migrate to the support surface and take part in the catalytic reaction of substance adsorbed on that site [136,137]. There are two conditions ensuring the HSPE can occur. First, there is the existence of metals capable of adsorbing dissociated hydrogen to convert hydrogen molecules into hydrogen atoms or hydrogen ions. Second, there is the presence of an acceptor for active hydrogen species and a channel and driver for the transfer of reactive hydrogen species [138]. The discovery of the HSPE has opened a new strategy in designing efficient catalysts. Especially, the HSPE has attracted much attention as one of the most potential technologies for enhancing the hydrogen storage performance of porous materials including MOFs at ambient temperature. The capacity of materials with induced hydrogen spillover was approximately five times higher than that of pristine MOFs [139]. Yang and co-workers doped 10 wt% Pt/AC catalysts into isoreticular MOF (IRMOF) materials to form a bridging spillover structure between carbon bridges and hydrogen spillovers, leading to a significant enhancement in the hydrogen storage performance. At 298 K and 100 bar, the hydrogen storage capacities of IRMOF-1 and IRMOF-8 increased from 0.4 to 3 wt% and from 0.5 to 4 wt%, respectively [140,141]. Table 7 summarizes the hydrogen storage capacities of some MOF materials.

### 2.3. Advantages and Disadvantages of Physical Hydrogen Storage Materials

Hydrogen storage technologies play an extremely important role in the “hydrogen economy”. It is necessary to continuously improve and develop storage materials for the hydrogen industry. As mentioned in the Introduction section, compared to chemical storage materials, the outstanding properties (Table 8) of the physical absorbents and the fact that they do not release greenhouse gases facilitate the global energy transition proceeding effectively as well as achieving the global “net zero emission” target sooner. However, each physical material has both its strength and weakness. The limitations of these materials presented in Table 8 are still challenges in practical hydrogen storage applications.

## 3. Conclusions and Recommendation

Materials for physical hydrogen storage such as hollow spheres, carbon-based materials, zeolites, and MOFs exhibit high hydrogen storage densities at cryogenic temperatures. Due to their limitation, they are capable of being utilized only at relatively low temperatures; thus, physical hydrogen storage materials are difficult to use in practical applications compared with that of chemical hydrogen storage materials such as aminoborane complex, liquid organic hydrogen, and metal hydrides at present. Significant efforts have been made to overcome these limitations. For example, certain carbon-based materials, zeolites, and modified MOFs exhibit significantly enhanced hydrogen densities at ambient temperatures. Still, to address these drawbacks and meet the requirements of commercial applications, further research and technical developments are necessary to obtain higher volumetric and gravimetric hydrogen densities. Nevertheless, the effort in developing next-generation materials for physical hydrogen storage should be continued to achieve fast and safe storage of the pure form of hydrogen. In order to open the hydrogen energy-based era in the future, various methodological combinations, as well as existing chemical hydrogen storage methods, will inevitably be required. Then, this physical hydrogen storage material and related methodologies are expected to be in the spotlight as an essential building block.

## Figures and Tables

**Table 1:** Hollow carbon sphere (HCS) synthesis methods.

Method	Type	Example Scheme
Hard templating	Silica template	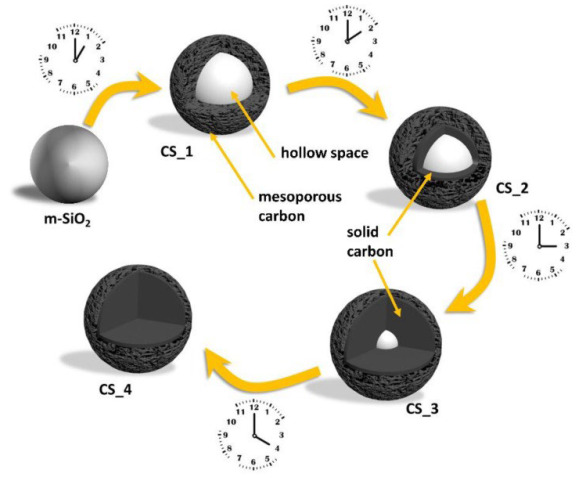 Scheme 1. Schematic of the formation process of hollow carbon spheres. Reprinted with permission from [25]. Copyright 2018, MDPI.
	Polymer template	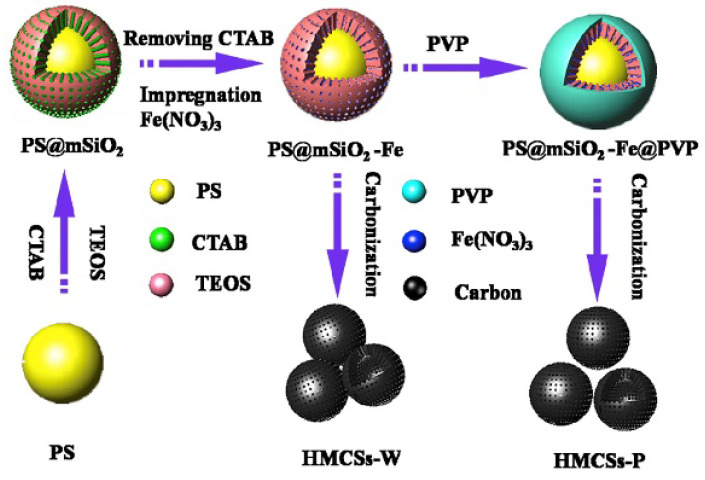 Scheme 2. Illustration of the synthesis route for hollow mesoporous carbon spheres. Reprinted with permission from [26]. Copyright 2018, Elsevier.
	Metal template	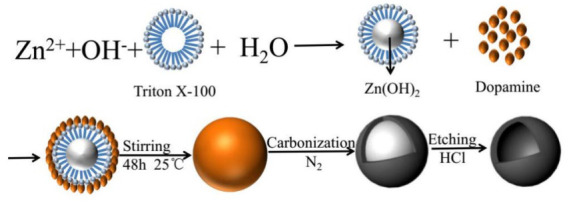 Scheme 3. Schematic of the synthetic process for hollow carbon spheres. Reprinted with permission from [27]. Copyright 2019, Elsevier.
Soft templating	Emulsion template	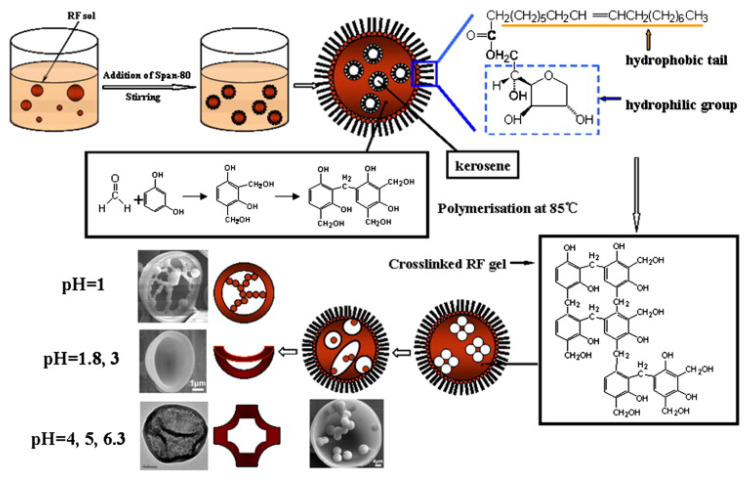 Scheme 4. Synthesis scheme for carbon hollow particles. Reprinted with permission from [28]. Copyright 2010, Elsevier.
	Surfactant template	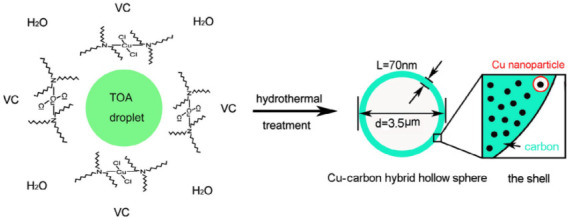 Scheme 5. Formation mechanism of Cu–C hybrid hollow spheres. Reprinted with permission from [29]. Copyright 2013, Elsevier.

**Table 2:** Various types of HCS used for hydrogen storage.

	Materials	Temperature (°C)	Pressure (bar)	H_2_ Storage Capacity (wt%)	Ave. Particle Size (nm)	Ave. Pore Size (nm)	Total Pore Volume (cm^3^/g)	Surface Area (m^2^/g)	Reference
1	Pd-hollow carbon spheres	40	24	0.36	~250	n/a	0.2514–0.9754	147–617	[33]
2	Necklace-like hollow carbon nanospheres (CNS)			0.89	60	5	n/a	594.32	[30]
3	Ni-decorated hollow carbon spheres	25	90	1.23	5100	n/a	0.31	28.6	[34]
4	Hollow nitrogen-containing carbon spheres (N-HCS)	−196	80	1.03	~250	1.38–20	0.84	872	[35]
		25	80	2.21					
5	Fe-nanoparticle–loaded hollow carbon spheres	300	20	5.6	200–500	~90	n/a	160	[36]
6	Metallic Mg ions diffused in hollow carbon nanospheres (HCNS)	270		6.78	440–9800	28	n/a	1810	[37]
		370		7.85					
7	Zeolite-like hollow carbon	−196	1	2.6	~5000	0.6–0.8	~2.41	3200	[38]
		−196	20	8.33					

**Table 3:** Summary of characterization of glass microspheres (GMs).

Preparation Method	Types	Advantages	Drawbacks
- Flame synthesis - Liquid droplet - Dried gel - Electrical arc plasma	- Solid GM - Hollow GM - Porous GM - Doped GM	- Excellent chemical stability - Chemical resistance - Strong mechanical strength - Low density - High thermal stability - High water resistance - Noncombustibility - Nonexplosibility - Nontoxicity - Fair cost	- Harsh synthetic condition - Fragility: weakness to impact damage - Not enough compressive strength - Poor thermal conductivity - Relatively difficult morphological control - Temperature sensitivity in hydrogen storage - Limited hydrogen storage capacity

**Table 4:** Carbon-based materials used in hydrogen storage.

Material	Definition	Advantages	wt% H_2_	T (K)	P (bar)	Ref.
Activated carbon (AC)	Modified synthetic carbon consisting of high-surface-area amorphous carbon and graphite; fabricated through thermal or chemical procedures [67]	High specific surface area Mechanical and chemical stabilities Microporous structure Relatively low cost Feasible commercial scaling [68,69]	0.1	298	10	[70]
			2.02	77		
			0.6	298	120	[71]
			4	77		
			0.85	77	100	[72]
			1.2	298	200	[73]
			2.7		500	
			5.6	77	40	
			1.09–2.05	298	0.00011	[74]
			5	77	30–60	[75]
Carbon nanotubes (CNTs)	CNTs are macromolecules containing a hexagonal arrangement of hybridized carbon atoms, which may be formed by rolling up a single sheet of graphene to form single-walled nanotubes (SWNTs) or multiple sheets of graphene to form multiwalled nanotubes (MWNTs) [76]	Unique structure Narrow size distribution and pore volume High surface area High strength Good electrical conductivity Good mechanical and thermal properties Low density Chemical stability Special functional properties [77,78,79,80]	SWNT			
			0.8		30	[81]
			1.73	77	100	[82]
			4.77	323		[83]
			4.77	323		[84]
			MWNT			
			0.2	298	100	[85]
			0.54	77	10	[86]
			1.7	298	120	[87]
			2		0.05	[88]
			3.46		127.9	[89]
			3.8	425	30	[90]
Graphite nanofibers (GNFs)	GNFs are produced from the dissociation of carbon-containing gases over a catalyst surface (e.g., metal or alloy) through chemical deposition. The solid consists of very small graphite platelets of width 30–500 Å, stacked in a perfectly arranged conformation [91]	Herringbone structure High degree of defects (exhibits the best performance for hydrogen storage) Several pretreatment procedures: oxidative, reductive, and inert environments [92]	1	300	20	[93]
			1.2	77	20	[94]
			3.3	298	48.3	[95]
			4–6.5	298	121.6	[96]
			1.3–7.5	298	100	[97]
			10–15	300	121.16	[98]
			7–10 (irreversible)			
			20–30 (reversible)			
Graphene-based materials	Graphene is a monolayer while graphite is a multilayer of carbon atoms strongly bound in a hexagonal crystal lattice. This is a carbon allotrope in the sp^2^ hybridized form with a molecular bond length of 0.142 nm [99]	High specific surface area High mechanical strength High corrosive-environment resistance High thermal and electrical conductivities [100,101] Various oxygen-containing functional groups	0.055	293	1.06	[102]
			0.13			
			0.14	298	1	[103]
			1.18		60	
			2.2	298	100	[104]
			3.3	323		[105]
			4.3	298	40	[106]
			1	293	120	
			5	77		
			6.28	298	1.01	[107]
			10.5	77	10	[108]

**Table 5:** Examples of hydrogen storage mechanisms of different types of carbon materials.

Carbon Material	Hydrogen Storage Mechanism
Activated carbon	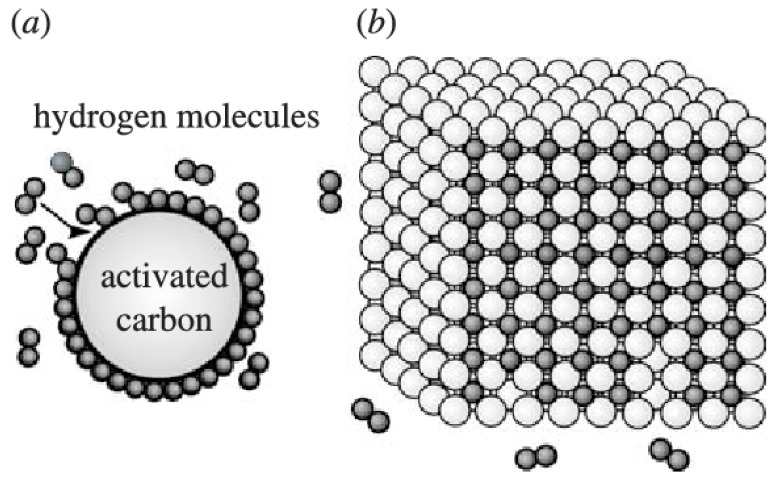 Scheme 6. Schematic of hydrogen (a) physisorption and (b) chemisorption using activated carbon (AC). Reprinted with permission from [109]. Copyright 2007, Royal Society
Carbon nanotube	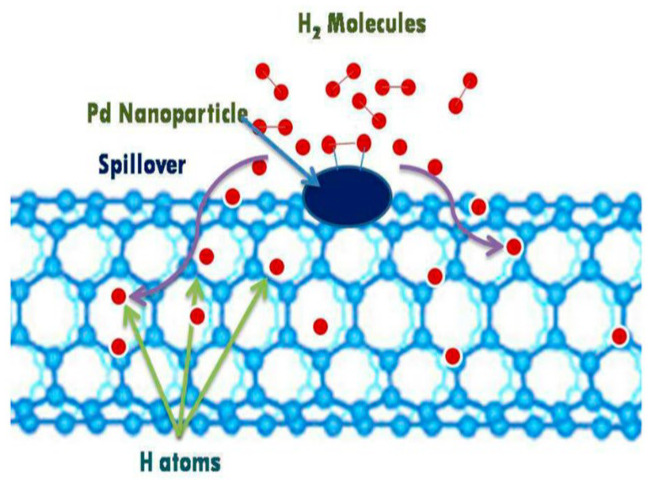 Scheme 7. Mechanism of carbon nanotube (CNTs) for hydrogen storage. Reprinted with permission from [110]. Copyright 2020, MDPI
Carbon nanofibrous	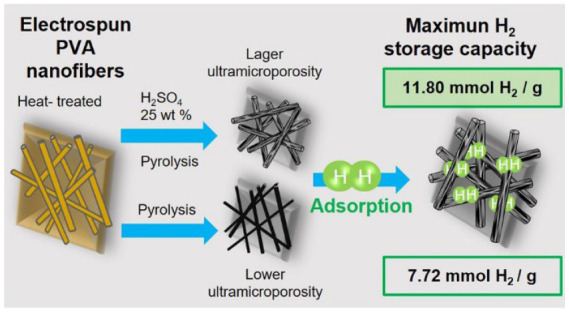 Scheme 8. Ultramicroporous carbon nanofibrous mats for hydrogen storage. Reprinted with permission from [111]. Copyright 2022, American Chemical Society
Graphene	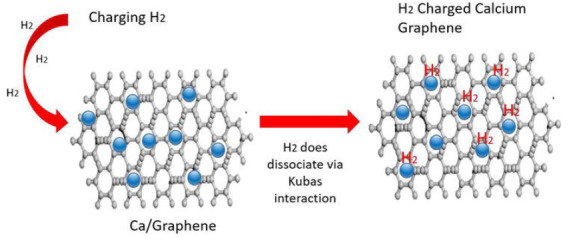 Scheme 9. (D) Reaction mechanism of hydrogen charging Ca/Graphene nanocomposite. Reprinted with permission from [112]. Copyright 2020, Intechopen

**Table 6:** Various synthesis and powder-shaping methods for metal–organic framework (MOF) materials.

MOF Synthesis Methods	MOF Powder-Shaping Methods
1. Microwave assisted method	1. Uniaxial pressing
2. Sonochemitry	2. Coating
3. Electrochemistry	3. Foaming
4. Mechanochemistry	4. Templating
5. Hydrothermal approach	5. Casting
6. Solvothermal approach	6. Granulation
	7. Extrusion
	8. Pulsed current processing

**Table 7:** Summary of hydrogen storage capacities of various MOF materials.

MOF Materials Used for Hydrogen Storage at Low Temperature				
Material	Temperature (K)	Pressure (bar)	H_2_ Storage Capacity (wt%)	Reference
Co-MOF	77	1	1.62	[132]
MIL-53	77	60	1.92	[129]
MIL-101 (Cr)/ZTC	77	1	2.55	[130]
CAU-1	70	1	4	
UBMOF-31	77	60	4.9	[131]
Fe-BTT	77	Low Pressure	2.3	[142]
	87		1.6	
	77	95	4.1	
NOTT-400	77	1	2.14	[143]
		20	3.84	
NOTT-401	77	1	2.31	
		20	4.44	
NU-1101	77–160	100–5	9.1	[144]
NU-1102	77–160	100–5	9.6	
NU-1103	77–160	100–5	12.6	
NPF-200	77	100–5	8.7	[145]
NU-1500	77–160	100–5	8.2	[146]
NU-1501-Al	77–160	100–5	14	[133]
MFU-4/Li	77–160	100–5	9.4	[134]
MOF materials used for hydrogen storage at ambient temperature				
Material	Temperature (K)	Pressure (bar)	H_2_ storage capacity (wt%)	Reference
HKUST-1	298	65	0.35	[147]
Cu-BTC	303	35	0.47	[148]
HKUST-1	303	35	0.47	[149]
CB/Pt/MOF-5	298	100	0.62	[150]
Zn_2_ (dobpdc)MOFs	298	100	1.3	[151]
Mg_2_ (dobpdc)MOFs	298	100	1.8	
Pd-CNMS/MOF-5	298	100	1.8	[152]
		75	1.6	
		50	1.4	
Be-BTB	298	100	2.3	[135]
V_2_Cl_2_._8_	298	100	1.64	[153]
NU-150I-Al	296	100	2.9	
IRMOF-1	298	100	3	[140]
IRMOF-8	298	100	4	[120]

**Table 8:** Advantages and disadvantages of physical hydrogen storage materials.

	Property	Prospects	Consequences
Material			
Hollow spheres		- High surface areas - High binding sites - High gravimetric energy density - Inexpensive manufacturing process - Safety - Scalable possibility	- Cryogenic temperatures - Agglomeration - Low volumetric energy density - Heating requirements for loading and unloading - Energy cost
Carbon-based materials		- Chemical stability - Good thermal - Simple processibility - Non-expensive	- Pore size uniformity - Binding site deficiency - Pore structure control
Zeolites		- Good crystallinity - Hydrolytic and thermal stability - Non-expensive	- Binding site deficiency - Low gravimetric loading - Tunability - Structural diversity limitation
MOFs		- High surface area - High pore volume - Good crystallinity - Tunability - Design control possibility - Rich metal sites	- Low hydrogen loading at room temperature - Fabrication process

## Data Availability

No new data were created.
